# Correction: Titov, E. On the Low-Lying Electronically Excited States of Azobenzene Dimers: Transition Density Matrix Analysis. *Molecules* 2021, *26*, 4245

**DOI:** 10.3390/molecules28031370

**Published:** 2023-02-01

**Authors:** Evgenii Titov

**Affiliations:** Theoretical Chemistry, Institute of Chemistry, University of Potsdam, Karl-Liebknecht-Straße 24-25, 14476 Potsdam, Germany; titov@uni-potsdam.de

During the course of a review of our publication, we found two errors in the Figure 3 Caption and Conclusions. We wish to make the following corrections to this paper [1].

**Figure Caption**

In the original publication, there was a mistake in the figure caption for **Figure 3.** FTDM matrices ^1^**F**,…,^5^**F** for the *S*_0_→*S*_1_ transition of azobenzene calculated with nine methods indicated in the top. FTDM elements are expressed in %, i.e., multiplied by 100. The correct figure caption appears below.

**Figure 3.** FTDM matrices ^1^**F**,…,^5^**F** for the *S*_0_→*S*_2_ transition of azobenzene calculated with nine methods indicated in the top. FTDM elements are expressed in %, i.e., multiplied by 100.

**Text Correction in Conclusions**

In the original publication, there was a mistake in “fraction of transition density” (FTDM) as published. The corrected version is “fraction of transition density matrix” (FTDM). 

The authors state that the scientific conclusions are unaffected. This correction was approved by the Academic Editor. The original publication has also been updated.

## References

[1] Titov E. (2021). On the Low-Lying Electronically Excited States of Azobenzene Dimers: Transition Density Matrix Analysis. Molecules.

