# Supplementary material for: On the Low-Lying Electronically Excited States of Azobenzene Dimers: Transition Density Matrix Analysis
Source: Molecules. 2021 Jul 13;26(14):4245. doi: 10.3390/molecules26144245 (PMC8303869; doi:10.3390/molecules26144245)
Supplement: Supplementary file 1 [file molecules-26-04245-s001.zip › Molecules_SI.pdf]

Supporting Information for

On the low-lying electronically excited states of azobenzene dimers: Transition density matrix analysis

Evgenii Titov*

Theoretical Chemistry, Institute of Chemistry, University of Potsdam, Karl-Liebknecht-Straße 24-25, 14476 Potsdam, Germany

*titov@uni-potsdam.de

Table S1: Vertical excitation energies in eV and oscillator strengths (in parentheses) of the lowest ten electronic transitions of the cofacial π-stacked azobenzene dimer d = 3.5 Å calculated with TD-ωB97X-D/6-31G*, TD-ωB97X-D/def2-TZVP, TD-ωB97X-D/aug-cc-pVTZ, and ADC(2)/aug-cc-pVTZ. The dimer geometry is constructed from the B3LYP/def2-TZVP optimized monomer geometry. The brightest transitions are shown in bold.

	TD-ωB97X-D/6-31G*	TD-ωB97X-D/def2-TZVP	TD-ωB97X-D/aug-cc-pVTZ	ADC(2)/aug-cc-pVTZ
$S_0 \rightarrow S_1$	2.59 (0.00)	2.57 (0.00)	2.57 (0.00)	2.65 (0.00)
$S_0 \rightarrow S_2$	2.63 (0.00)	2.63 (0.00)	2.63 (0.00)	2.72 (0.00)
$S_0 \rightarrow S_3$	3.58 (0.00)	3.45 (0.00)	3.43 (0.00)	3.46 (0.00)
$S_0 \rightarrow S_4$	**4.32 (1.40)**	**4.21 (1.37)**	**4.19 (1.36)**	4.04 (0.00)
$S_0 \rightarrow S_5$	4.39 (0.00)	4.27 (0.00)	4.25 (0.00)	4.04 (0.00)
$S_0 \rightarrow S_6$	4.40 (0.00)	4.28 (0.00)	4.27 (0.00)	**4.20 (1.50)**
$S_0 \rightarrow S_7$	4.64 (0.00)	4.59 (0.00)	4.58 (0.00)	4.47 (0.04)
$S_0 \rightarrow S_8$	4.69 (0.00)	4.66 (0.00)	4.66 (0.00)	4.48 (0.00)
$S_0 \rightarrow S_9$	4.73 (0.01)	4.67 (0.00)	4.67 (0.00)	4.51 (0.00)
$S_0 \rightarrow S_{10}$	4.81 (0.05)	4.73 (0.04)	4.71 (0.04)	4.56 (0.00)

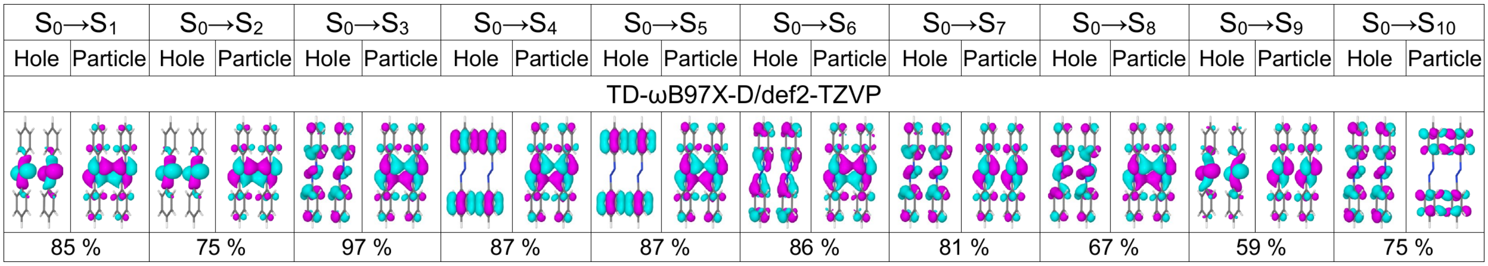

Figure S1: Dominant natural transition orbital pairs for the lowest ten transitions of the π-stacked dimer d = 3.0 Å. Calculations are performed at the TD-ωB97X-D/def2-TZVP level of theory.

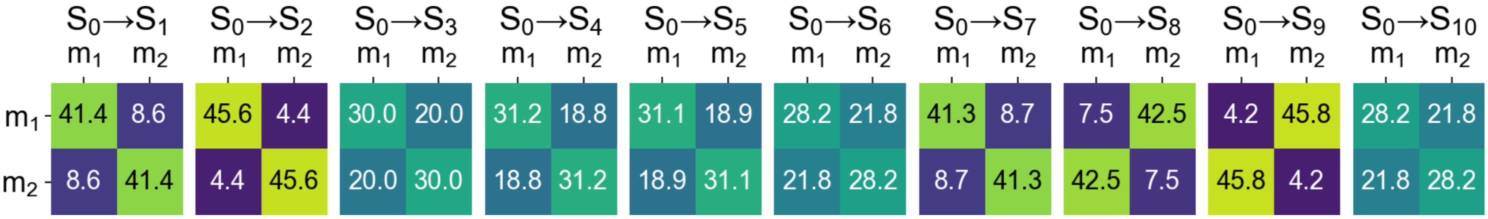

Figure S2: FTDM $^5\mathbf{F}$ matrices for the lowest ten transitions of the π-stacked dimer d = 3.0 Å. Calculations are performed at the TD-ωB97X-D/def2-TZVP level of theory.
